# Defective Hand1 phosphoregulation uncovers essential roles for Hand1 in limb morphogenesis

**DOI:** 10.1242/dev.149963

**Published:** 2017-07-01

**Authors:** Beth A. Firulli, Hannah Milliar, Kevin P. Toolan, Jade Harkin, Robyn K. Fuchs, Alex G. Robling, Anthony B. Firulli

**Affiliations:** 1 Riley Heart Research Center, Herman B Wells Center for Pediatric Research, Departments of Anatomy and Cell Biology, Biochemistry, Medical and Molecular Genetics, Indiana University School of Medicine; 2 Department of Physical Therapy and the Center for Translational Musculoskeletal Research, School of Health and Rehabilitation Science, Indiana University, Indianapolis, IN 46202, USA; 3 Department of Anatomy and Cell Biology, Indiana University School of Medicine, 635 Barnhill Drive, Indianapolis, IN 46202-5225, USA

**Keywords:** Hand1, bHLH, Dimerization, Limb development, Transcription, Mouse

## Abstract

The morphogenesis of the vertebrate limbs is a complex process in which cell signaling and transcriptional regulation coordinate diverse structural adaptations in diverse species. In this study, we examine the consequences of altering Hand1 dimer choice regulation within developing vertebrate limbs. Although *Hand1* deletion via the limb-specific *Prrx1-Cre* reveals a non-essential role for Hand1 in mouse limb morphogenesis, altering Hand1 phosphoregulation, and consequently Hand1 dimerization affinities, results in a severe truncation of proximal-anterior limb elements. Molecular analysis reveals a non-cell-autonomous mechanism that causes widespread cell death within the embryonic limb bud. In addition, we observe changes in proximal-anterior gene regulation, including a reduction in the expression of *Irx3*, *Irx5*, *Gli3* and *Alx4*, all of which are upregulated in *Hand2* limb conditional knockouts. A reduction of *Hand2* and *Shh* gene dosage improves the integrity of anterior limb structures, validating the importance of the Twist-family bHLH dimer pool in limb morphogenesis.

## Abstract (precis)

**Summary:** Altering Hand1 phosphoregulation, and consequently Hand1 dimerization affinities, results in a severe truncation of anterior-proximal limb elements in mice.

## INTRODUCTION

The vertebrate limb is an evolutionarily dynamic structure that has adapted broadly to fit the intended functional role, be it running, climbing, flying or swimming. As a consequence of this adaptability, defects in limb morphogenesis are common due to the complexities of limb development, where subtle temporal and/or spatial changes in gene regulation can result in a multitude of limb defects ranging from mild to severe. In humans, forelimb congenital defects occur at a frequency of 1 in 500 live births (Furniss et al., 2009; Zuniga et al., 2012). Moreover, 18% of children born with congenital limb defects die by 6 years of age due to additional associated defects in more vital organs. Although the mechanisms of limb outgrowth and patterning are well defined, a better understanding of the underlying relationships between the gene programs causative of proximal limb deformities could facilitate a better understanding of how these same programs interplay in other organ systems resulting in defects that lead to lethality.

Members of the Twist family of bHLH transcription factors play a crucial role in patterning of the limb (Barnes and Firulli, 2009; Firulli et al., 2005, 2007; Galli et al., 2010; Krawchuk et al., 2010; Loebel et al., 2014; O'Rourke et al., 2002; Osterwalder et al., 2014; te Welscher et al., 2002). Systemic loss of *Twist1* results in mid-gestation lethality that is accompanied by hypoplastic limbs (Chen and Behringer, 1995), while *Twist1* heterozygous mice exhibit a partially penetrant preaxial polydactyly modeling Saethre-Chotzen syndrome (SCS) that can be rescued by a gene dosage reduction in *Hand2* (a Twist1 antagonist; Firulli et al., 2005). *Twist1* expression marks the mesoderm underlining the apical ectodermal ridge (AER). Twist1, when temporally deleted, results in proximal and preaxial anatomical phenotypes in the limbs (Loebel et al., 2012, 2014). Hand2 plays a key role in the gene pathways that are essential for setting up the proximal, posterior and anterior limb bud gene regulatory networks via regulation of key limb patterning factors that include *Tbx3*, *Gli3*, *Irx3/5* and *Shh* (Galli et al., 2010; Osterwalder et al., 2014).

The Twist family member *Hand1* is also expressed within the developing limb (Fernandez-Teran et al., 2003) and the overexpression of *Hand1* within limb mesoderm results in preaxial polydactyly via increased *Shh* expression, similar to Hand2 limb gain-of-function (Fernandez-Teran et al., 2003; McFadden et al., 2002). Recently, Hand1 has been implicated in endochondral ossification of the cartilage primordia via a gain-of-function analysis (Laurie et al., 2016); however, little else is understood about the role of Hand1 in limb development. It is well established that Twist family bHLH factors such as Hand1 exhibit homodimerization and heterodimerization with other bHLH factors outside of E-proteins. Dimerization of Twist family proteins is regulated by phosphorylation of highly conserved threonine and serine residues within helix I of the bHLH domain present in all family members (Firulli et al., 2000, 2005, 2007, 2014). In SCS, several *TWIST1* mutations causative of SCS alter TWIST1 phosphoregulation and dimerization characteristics (Firulli et al., 2005). In trophoblast giant cells, Hand1 phosphoregulation modulates its nuclear localization, which dictates cell differentiation (Martindill et al., 2007), and when Hand1 dimer mutants are expressed within postmigratory cranial neural crest, large-scale craniofacial defects are encountered (Firulli et al., 2014).

Here, we explore the role of Hand1 in mouse limb morphogenesis using loss-of-function and gain-of-function analyses. We find that *Prrx1-Cre*-mediated deletion of *Hand1* confers no observable limb abnormalities. We next employed our knock-in *Hand1* phospho-mutant alleles to investigate the role of Hand1 dimer regulation in limb morphogenesis. Hand1 mutants exhibit abnormal limb development of anterior structures accompanied by widespread cell death within the limb buds. Additionally, the downregulation of key genes required for proximal-anterior identity, including the established Hand2 transcriptional targets *Irx3*, *Irx5 Alx4* and *Gli3*, is observed. Finally, we partially restore anterior limb structures by reducing the gene dosage of *Hand2* and *Shh*, revealing that the balance between posterior and anterior limb structures is dependent on Twist family dimer choices during limb development.

## RESULTS

### Deletion of *Hand1* within the forming limb mesoderm has no effect on morphogenic patterning

To investigate the role of Hand1 in limb formation, we crossed *Hand1* conditional knockout mice (McFadden et al., 2005) with the limb-specific *Prrx1-Cre* transgenic line (Logan et al., 2002). Mice were born at the expected mendelian ratios and open examination of shoulder, hip and limb structures showed *Hand1* conditionally deleted mice to be indistinguishable from wild-type littermate controls (data not shown). Although Hand1 appears to play no necessary role in limb morphogenesis when deleted with *Prrx1-Cre*, we reasoned that its participation in regulating the bHLH dimer pool within the cells of the developing limb bud could result in disruption of limb morphogenesis, as suggested from observations comparing *Hand1* deletion within the neural crest versus disruption of Hand1 dimerization (Barbosa et al., 2007; Firulli et al., 2014).

We first examined transgenic mice that overexpressed *Hand1* via the 2.4 kb *Prrx1* limb enhancer (Martin and Olson, 2000) (Fig. S1). P0 neonates were examined for the presence of polydactyly, as previously reported for Hand gain-of-function (Charite et al., 2000; Fernandez-Teran et al., 2000; McFadden et al., 2002; te Welscher et al., 2002). In addition to polydactyly, several neonates exhibited severe loss of one or both forelimbs and/or hindlimbs, reflecting a clear limitation of transgenic expression analysis, which could be saturating the bHLH dimer pool, disrupting anteroposterior polarity and/or abrogating cell signaling (Fig. S1), limiting our ability to assess Hand1 dimer regulation. Of note, this observed loss of limb structure in *Prrx1-Hand1* gain-of-function transgenics is similar to that observed in *Hand2* limb loss-of-function analysis (Galli et al., 2010).

### Hand1 phosphorylation mutants display proximal-anterior limb phenotypes

**Fig. 1.**
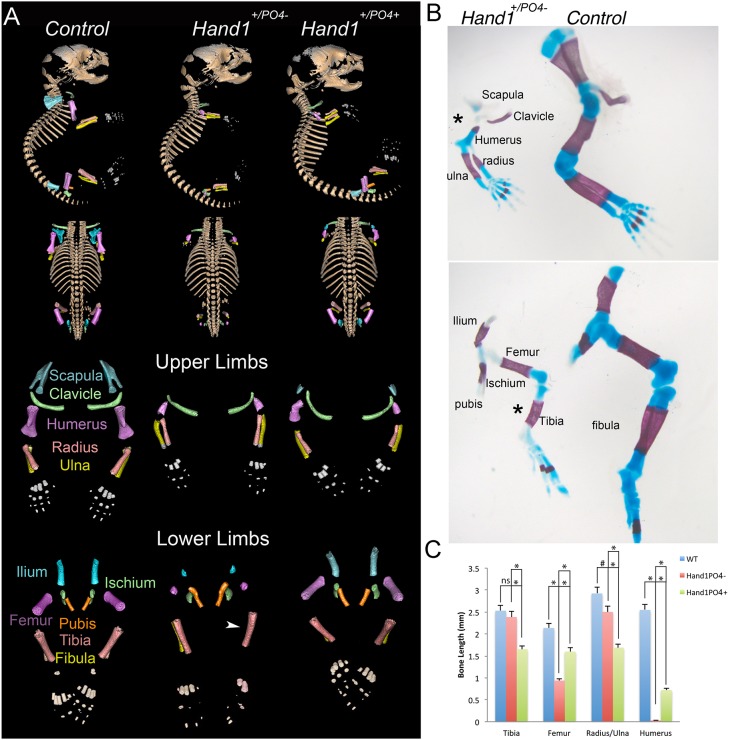
**Proximal limb abnormalities in *Prrx1-Cre;Hand1^+/PO4−^* and *Prrx1-Cre;Hand1^+/PO4+^* mice.** (A) Micro-CT analysis of control (left column), Hand1 hypophosphorylation mutant (middle column) and Hand1 phosphorylation mimic (right column) P0 neonatal mice. Examination of forelimbs reveals a significant reduction in scapula (cyan) and humerus (purple), whereas radius (red) and ulna (yellow) are less affected but significantly smaller and less well developed than control bones. Hindlimb examination shows that ilium (blue), femur (purple), tibia and fibula (red and yellow, respectively) are also affected. (B) Skeletal preparation of a *Prrx1-Cre;Hand1^+/PO4−^* neonate shows that the level of ossification is greatly reduced in mutant compared with control fore- and hindlimbs. Arrowhead (A) and asterisks (B) indicate the missing fibula encountered in *Prrx1-Cre;Hand1^+/PO4−^* hindlimbs with incomplete penetrance. (C) Measurements taken from five examples of control (WT), *Prrx1-Cre;Hand1^+/PO4−^* and *Prrx1-Cre;Hand1^+/PO4+^* via micro-CT reveal significant reductions in the size of femurs, humerus and radius/ulna in *Prrx1-Cre;Hand1^+/PO4−^* mutants and significant reductions in the size of femur, tibia, humerus and radius/ulna in *Prrx1-Cre;Hand1^+/PO4+^* mutants (*P≤0.001, ^#^*P*≤0.05). Significant differences in the sizes of tibia, femur, humerus and radius/ulna are also observed between *Prrx1-Cre;Hand1^+/PO4^* and *Prrx1-Cre;Hand1^+/PO4+^* neonates. ns, not significant. Error bars indicate standard error.

To test Hand1 gain-of-function more rigorously in the limbs, we used *Prrx1-Cre* to activate the expression of conditional *Hand1* hypophosphorylation (*Hand1^PO4^*^−^) and phosphorylation mimic (*Hand1^PO4^*^+^) alleles that are knocked into the endogenous *Hand1* locus (Firulli et al., 2014), activating *Hand1* mutant allelic expression within only *Hand1*-expressing limb tissue at endogenous levels. Hand1 dimer choice and activity are in part regulated by the phosphorylation of conserved threonine 107 and serine 109 within the first helix of the bHLH domain (Firulli et al., 2003). *Hand1^PO4^*^−^ and *Hand1^PO4^*^+^ are targeted conditional knock-in alleles that act as a *Hand1* null allele until the Stop-Flox cassette within the *Hand1* 5′UTR is efficiently removed by Cre recombinase (Firulli et al., 2014). *Prrx1-Cre* is expressed throughout the forming fore- and hindlimbs by E10.5, including the underlying lateral mesoderm (Logan et al., 2002) (Fig. S2). The intersection of *Prrx1-Cre* activity and *Hand1* endogenous expression is where these knock-in alleles will be expressed at levels comparable to endogenous *Hand1* expression. P0 *Prrx1-Cre;Hand1^+/PO4−^* and *Prrx1-Cre;Hand1^+/PO4+^* neonates were obtained at mendelian ratios; however, they exhibited severe limb defects within the stylopods and zeugopods for both fore- and hindlimbs (Fig. 1). Micro-CT scans of P0 neonate forelimbs revealed the absence of a mineralized scapula (a derivative of lateral mesoderm) in *Prrx1-Cre;Hand1^+/PO4−^* neonates when compared with controls (Fig. 1A). In *Prrx1-Cre;Hand1^+/PO4+^* neonates, a mineralized scapula is present but significantly reduced in size. Skeletal staining confirms the lack of mineralized scapula in *Prrx1-Cre;Hand1^+/PO4−^* neonates (Fig. 1B). The size of the humerus is also significantly reduced in both *Prrx1-Cre;Hand1^+/PO4−^* and *Prrx1-Cre;Hand1^+/PO4+^* neonates compared with controls (Fig. 1A,C). Moreover, the humerus in *Prrx1-Cre;Hand1^+/PO4−^* neonates is significantly smaller than in *Prrx1-Cre;Hand1^+/PO4+^* neonates (Fig. 1A,C). The radius and ulna of *Prrx1-Cre;Hand1^+/PO4−^* neonates are significantly reduced in size compared with controls (Fig. 1C); however, the radius and ulna of *Prrx1-Cre;Hand1^+/PO4+^* neonates is significantly smaller than those of both *Prrx1-Cre;Hand1^+/PO4−^* and control neonates (Fig. 1C). Clavicle size, shape and mineralization appear unchanged in Hand1 phospho-mutants compared with controls.

Hindlimb structures mirror what is observed in the forelimb. The ilium is smaller and displays less mineralization in *Prrx1-Cre;Hand1^+/PO4−^* than in control neonates, and this phenotype is less severe in *Prrx1-Cre;Hand1^+/PO4+^* mice (Fig. 1A). Femur size and mineralization are also significantly reduced in both *Prrx1-Cre;Hand1^+/PO4−^* and *Prrx1-Cre;Hand1^+/PO4+^* neonates compared with controls (Fig. 1C). The length and shape of the tibia are not significantly different between controls and *Prrx1-Cre;Hand1^+/PO4−^* neonates, but tibia length is significantly reduced in *Prrx1-Cre;Hand1^+/PO4+^* mice compared with both control and *Prrx1-Cre;Hand1^+/PO4−^* neonates (Fig. 1A,C). Interestingly, the missing fibula is encountered in *Prrx1-Cre;Hand1^+/PO4−^* hindlimbs with incomplete penetrance (Fig. 1A, arrowhead; Fig. 1B, asterisks). Pubis and ischium are not noticeably altered in Hand1 phospho-mutant mice. X-ray images of control, *Prrx1-Cre;Hand1^+/PO4−^* and *Prrx1-Cre;Hand1^+/PO4+^* mice support these observations (Fig. S3).

**Fig. 2.**
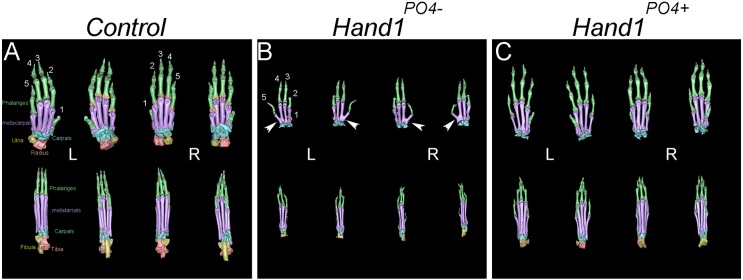
**Autopod abnormalities in *Prrx1-Cre;Hand1^+/PO4−^* and *Prrx1-Cre;Hand1^+/PO4+^* mice.** Left (L) and right (R) autopods from fore- and hindlimbs of (A) control, (B) *Prrx1-Cre;Hand1^+/PO4−^* and (C) *Prrx1-Cre;Hand1^+/PO4+^* neonatal mice. Phalanges (green), metacarpals (purple) and carpals (light blue) are shown both from dorsal and ventral views. Although largely normal in appearance, *Prrx1-Cre;Hand1^+/PO4−^* forelimbs are small and show fusion between digit 5 and 4 metacarpals (arrowheads). This fusion is not observed in *Prrx1-Cre;Hand1^+/PO4+^* forelimbs, which are of intermediate size compared with both controls and *Prrx1-Cre;Hand1^+/PO4−^* mice. *n*=5.

Examination of the micro-CT imaged autopods of *Prrx1-Cre;Hand1^+/PO4−^* and *Prrx1-Cre;Hand1^+/PO4+^* fore- and hindlimbs reveal smaller front and hind paws (Fig. 2). *Prrx1-Cre;Hand1^+/PO4−^* neonate forelimbs display a fusion of the digit 5 and 4 metacarpals (Fig. 2B) that is not observed in the hindlimbs or in the limbs of *Prrx1-Cre;Hand1^+/PO4+^* mice.

### Hand1 phospho-mutant phenotypes are associated with high levels of limb bud cell death

**Fig. 3.**
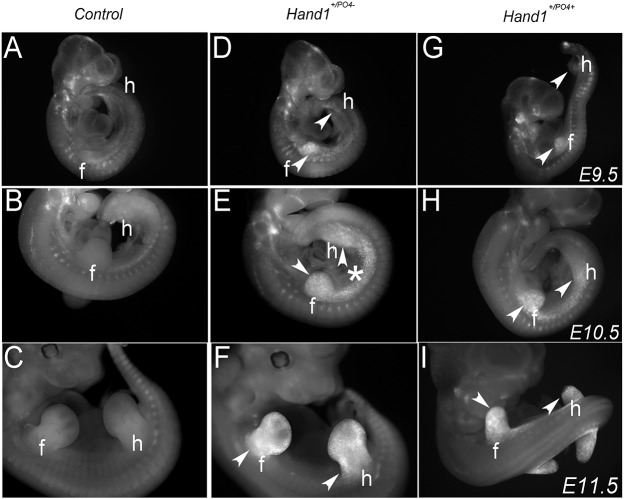
**Both cell-autonomous and non-cell-autonomous cell death is present in the developing limbs of Hand1 phospho-mutant mice.** Lysotracker staining of control (A-C), *Prrx1-Cre;Hand1^+/PO4−^* (D-F) and *Prrx1-Cre;Hand1^+/PO4+^* (G-I) embryos between E9.5 and E11.5, indicating forelimb (f) and hindlimb (h) cell death (arrowheads) during development. Compared with controls, expression of Hand1 phospho-mutant protein results in a considerable degree of cell death within fore- and hindlimbs as well as in the intervening lateral mesoderm. *n*=5. Asterisk indicates lateral mesoderm cell death.

When expressed within the neural crest, Hand1 phospho-mutants caused non-cell-autonomous cell death within the forming mandibular arch, altering gene expression within both the Fgf and Shh signaling pathways (Firulli et al., 2014). As it is clear that expression of Hand1 phospho-mutants in limb mesoderm results in hypoplastic limbs, we first looked at cell death in fore- and hindlimbs between E9.5 and E11.5 (Fig. 3). Compared with controls, both *Prrx1-Cre;Hand1^+/PO4−^* fore- and hindlimbs exhibited a marked increase in cell death between E9.5 and E11.5 as assayed by lysotracker staining (Fig. 3D-F). The dying cells within the limb buds represent both cell-autonomous (anterior-proximal and posterior-proximal limb bud) and non-cell-autonomous domains when compared with *Hand1* limb expression (Fig. S4A-D). Cell death is also markedly enhanced within the intervening lateral mesoderm posterior to the forelimb and anterior to the hindlimb, correlating with the Cre activity observed from the *Prrx1-Cre* transgene at E10.5 (Fig. S2). *Prrx1-Cre;Hand1^+/PO4+^* fore- and hindlimbs exhibit a similar pattern of cell death (Fig. 3G-I). No changes in cell proliferation were observed (data not shown), a finding consistent with observations of phospho-mutant expression in neural crest cells (Firulli et al., 2014).

### Expression analysis of Hand1 phospho-mutants reveals gene expression changes in Hand2 transcriptional targets

**Fig. 4.**
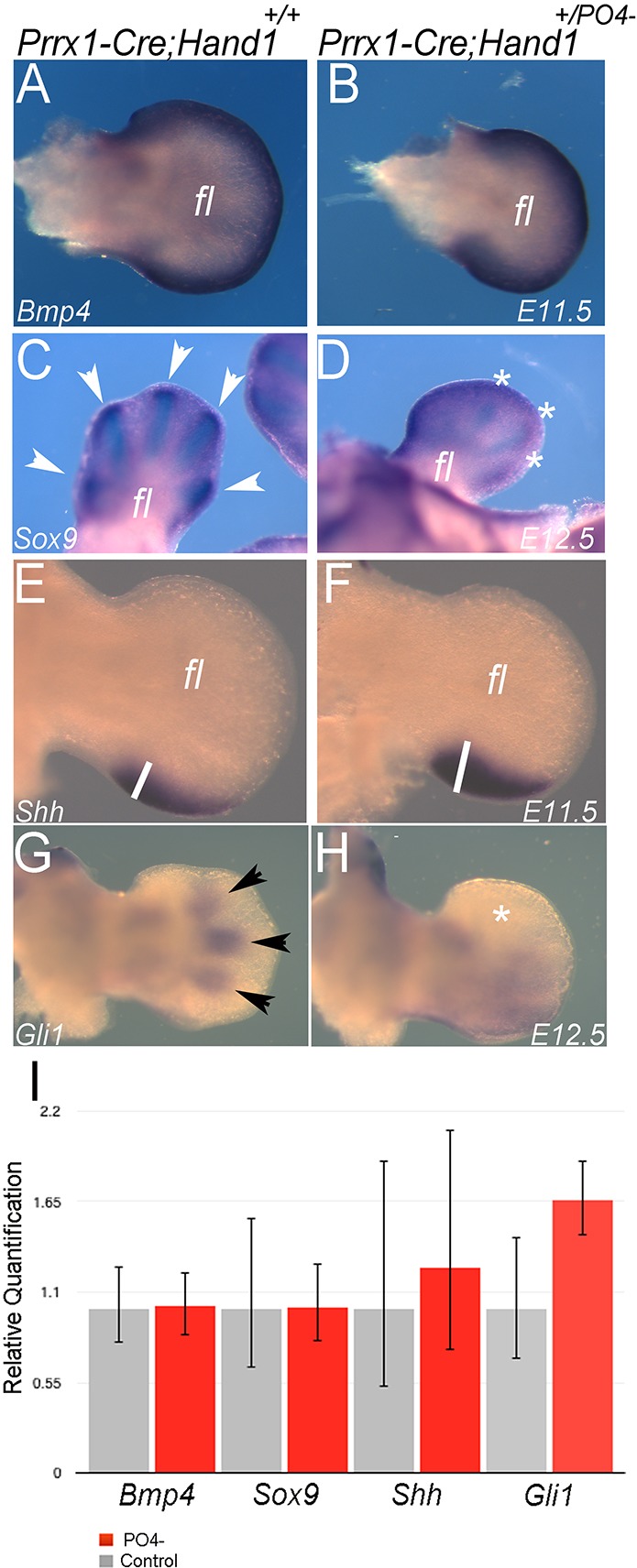
**Shh pathway gene expression is altered in *Prrx1-Cre;Hand1^+/PO4−^* mice.** (A-H) Whole-mount *in situ* hybridizations of forelimbs (fl) at E11.5 (A,B,E,F) and E12.5 (C,D,G,H) for the indicated markers in *Prrx1-Cre* controls and *Prrx1-Cre;Hand1^+/PO4−^* littermates. (A,B) *Bmp4* expression is unchanged in *Prrx1-Cre;Hand1^+/PO4−^* mutants. Arrowheads in C mark *Sox9* digit expression and the asterisks in D mark the degraded specificity of Sox9 digit expression in *Prrx1-Cre;Hand1^+/PO4−^* mutants. Lines in E and F measure the anterior reach of *Shh* expression. Arrowheads in G mark the forming digit expression of *Gli1* and the asterisk in H reflects the loss of this patterning but not of expression. (I) qRT-PCR analysis of E10.5 control and *Prrx1-Cre;Hand1^+/PO4−^* littermates indicates that there are no statistically significant differences in the expression of these markers by two-tailed *t*-test. Error bars represent the high and low range of replicate cycle reads within each primer set. *n*=6.

To begin to understand how Hand1 dimer mutants are altering limb morphogenesis, we first looked at the expression of *Bmp4* (which is crucial for early limb patterning and skeletogenesis). In E11.5 embryos, *Bmp4* expression appears indistinguishable between control and *Prrx1-Cre;Hand1^+/PO4−^* mutants, being evident within the underlying mesoderm proximal to the AER in the forelimbs (Fig. 4A,B). We next utilized the chondrocyte marker *Sox9* (Fig. 4C,D), which also shows equivalent expression by staining, but with reduced specificity within the forming digits (compare arrowheads with asterisks). Next, we looked at *Fgf8*, as it is essential for limb outgrowth via specification of the AER (Boulet and Capecchi, 2004; Chrisman et al., 2004; Yoon et al., 2000). Moreover, *Fgf8* expression is altered by Hand1 phospho-mutant expression within the cranial neural crest (Firulli, et al., 2014). We carefully monitored *Fgf8* expression between E9.5 and E12.5 in control and *Prrx1-Cre;Hand1^+/PO4−^* embryos and found no observable changes in expression (Fig. S5). Shh is also a key regulator of limb development and patterning required for establishing the zone of polarizing activity (ZPA) and is a direct transcriptional target of Hand2; in addition, *Shh* expression is altered by Hand1 phospho-mutant expression within the cranial neural crest (Firulli et al., 2014). *Shh* expression within the ZPA extends more anteriorly than in controls (compare line length in Fig. 4E,F), suggesting that Shh signaling is enhanced in *Prrx1-Cre;Hand1^+/PO4−^* embryos.

To probe the Shh pathway further, we looked at expression of the Shh signaling mediator *Gli1* at E12.5*.* Control forelimbs show discrete domains of *Gli1* expression within the forming digits (Fig. 4G), whereas *Prrx1-Cre;Hand1^+/PO4−^* mutant limbs show robust *Gli1* expression within the more posterior portion of the limb bud (Fig. 4H).

To quantify expression levels, similar forelimbs were collected, RNA isolated and used to generate cDNA for qRT-PCR analysis (Fig. 4I). Results show no significant difference in expression levels of the aforementioned genes, although the upregulation of *Gli1* is nearly significant (*P*≤0.06). These data suggest that although Shh pathway gene expression is not directly increased, its expression domain within the limb is expanded, and we sought to identify the alternative mechanism regulating this phenomenon.

We reasoned that Hand2, which is well established as an inducer of *Shh* (Fernandez-Teran et al., 2003; McFadden et al., 2002; te Welscher et al., 2002), or Twist1, a Hand2 antagonist during limb morphogenesis (Firulli et al., 2005), could exhibit altered expression within *Prrx1-Cre;Hand1^+/PO4−^* embryos. Expression of *Hand2* and *Twist1* in E12.5 *Prrx1-Cre;Hand1^+/PO4−^* mutant limbs was unaltered (Fig. S4E-L), and so we next investigated the expression of Hand2 transcriptional targets that control proximal-anterior limb patterning.

### Proximal-anterior genes inhibited by Hand2 are inhibited in Hand1 phospho-mutants

**Fig. 5.**
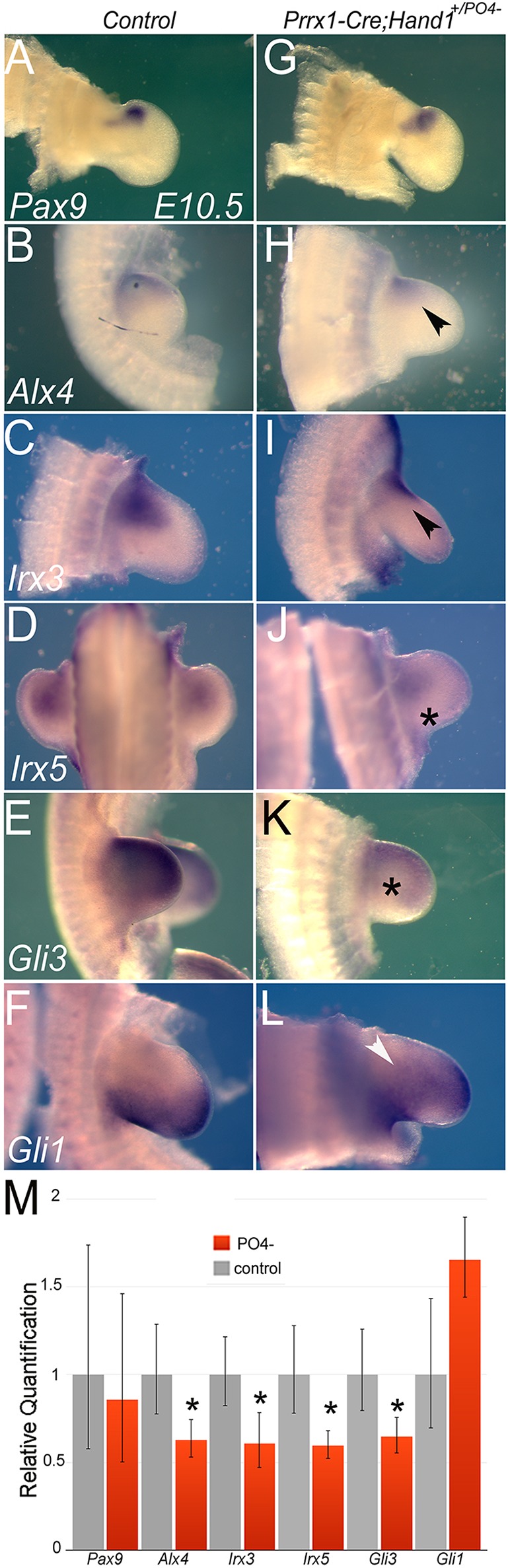
***Prrx1-Cre;Hand1^+/PO4−^* mutants show reductions in gene expression of factors defining proximal-anterior limb identity.** (A-L) Whole-mount *in situ* hybridizations of E10.5 forelimbs from control (*Prrx1-Cre*) and *Prrx1-Cre;Hand1^+/PO4−^* littermates. The proximal-anterior limb marker *Pax9* shows no significant changes in expression between mutant and control (A,G). By contrast, *Alx4* (B,H), *Irx3* (C,I), *Irx5* (D,J) and *Gli3* (E,K) show markedly reduced levels of expression (arrowheads and asterisks) in *Prrx1-Cre;Hand1^+/PO4−^*. *Gli1* expression (F,L) appears to expand anteriorly (arrowhead) with the limbs of *Prrx1-Cre;Hand1^+/PO4−^* mutants. (M) qRT-PCR analysis confirms significant downregulation (*P≤0.05, two-tailed *t*-test). *n*=6. Error bars indicate the high and low range of replicate cycle reads with cycle reads within each primer set.

To better understand the mechanism by which Hand1 dimer control was altering limb patterning and development, we looked to gene regulatory networks that modulate proximal-anterior structures. We first looked at the expression of the anterior domain marker *Pax9* (Fig. 5A,G), but found no significant difference in expression between *Prrx1-Cre* controls and *Prrx1-Cre;Hand1^+/PO4−^* mutants. By contrast, the anterior marker *Alx4* showed a marked reduction in expression in *Prrx1-Cre;Hand1^+/PO4−^* forelimbs compared with controls (Fig. 5B,H). Interestingly, *Alx4* is also upregulated within limb buds of Hand2 loss-of-function embryos (Osterwalder et al., 2014). Transcription factors Irx3 and Irx5 are required for the specification of humerus/femur and radius/tibia progenitors (Li et al., 2014). Moreover, *Irx3;Irx5* double-knockout mice exhibit a similar phenotype to Hand1 phospho-mutants and *Irx3* and *Irx5* are also upregulated in Hand2 loss-of-function mice (Osterwalder et al., 2014). Results show that expression of *Irx3* (Fig. 5C,I) and *Irx5* (Fig. 5D,J) is greatly reduced. Expression of the transcriptional repressor *Gli3* is also reduced in *Prrx1-Cre;Hand1^+/PO4−^* forelimbs compared with controls (Fig. 5E,K) and, accordingly, *Gli3* expression is upregulated in Hand2 loss-of-function mice (Osterwalder et al., 2014). Similar to observations in older limb buds (Fig. 4G,H), *Gli1* expression is expanded in *Prrx1-Cre;Hand1^+/PO4−^* mutant E10.5 forelimbs compared with controls (Fig. 5F,L). qRT-PCR analysis of RNA isolated from similar forelimbs confirmed the reductions observed in whole-mount analysis as statistically significant (Fig. 5M). A trend was also observed for an increase in *Gli1* expression, although this upregulation was not statistically significant (Fig. 5M; *P*=0.19).

### Reduction in *Hand1*, *Hand2* or *Shh* gene dosage improves the Hand1 phospho-mutant limb phenotype

**Fig. 6.**
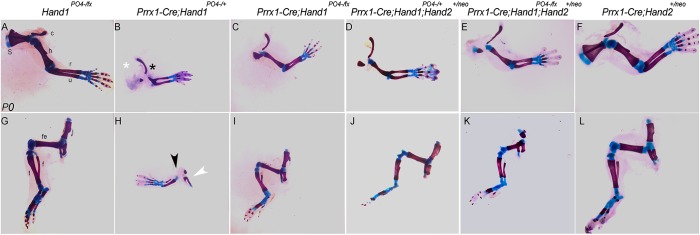
**Loss of wild-type *Hand1* and *Hand2* alleles improves limb development.** (A) P0 skeletal preparation of control forelimb showing normal bone formation. (B) Forelimb skeletal preparation showing loss/reduction of scapula (s, white asterisk) and humerus (h, black asterisk) in *Prrx1-Cre;Hand1^+/PO4−^* mice. (C) Deletion of the wild-type *Hand1* allele (*Prrx1-Cre;Hand1^fx/PO4−^*) partially restores these proximal-anterior bones. (D) Reduction of *Hand2* gene dosage also partially restores phenotype and (E) reduction of both wild-type *Hand1* and *Hand2* does not further restore limb structures. (F) No limb defects are observed in *Prrx1-Cre;Hand2^+/neo^* mice. (G) P0 skeletal preparation of control hindlimb showing normal bone formation. (H) Hindlimb skeletal preparation showing loss/reduction of ilium (i, white arrowhead) and femur (fe, black arrowhead) in *Prrx1-Cre;Hand1^+/PO4−^* mice. (I) Deletion of the wild-type *Hand1* allele (*Prrx1-Cre;Hand1^fx/PO4−^*) partially restores these proximal-anterior bones. (J) *Hand2* haploinsufficiency improves hindlimb morphology. (K) Reduction of both *Hand1* and *Hand2* does not further improve hindlimb and (L) *Prrx1-Cre;Hand2^+/neo^* mice exhibit normal hindlimb structures. *n*=5. c, clavicle; f, fibula; r, radius; t, tibia; u, ulna.

A key observation in our expression analysis is that several of the downregulated genes in the *Prrx1-Cre;Hand1^+/PO4−^* mutant limbs are upregulated in *Hand2*-deficient limbs. Although we observed no changes in *Hand1* or *Hand2* gene expression within the limb buds (Fig. S4), the possibly exists that our Hand1 dimer mutants were interacting with Hand2, enhancing its inhibitory regulation on proximal-anterior gene programs. We sought to test such interactions via genetic rescue (Fig. 6). We intercrossed *Prrx1-Cre;Hand1^+/PO4−^* mice onto either the conditional *Hand1* allele (*Hand1^fx^*; McFadden et al., 2005) or *Hand2^n^^eo^* allele (Srivastava, 1997) and interrogated limb structures in P0 skeletal preparations. *Prrx1-Cre;Hand1^+/PO4−^* limbs exhibit the observed limb phenotype (see Fig. 1B), with the loss of scapula and reduced humerus (Fig. 6A,B). In the hindlimb, both the ilium and femur are similarly reduced in size when compared with control (Fig. 6G,H). Deletion of the wild-type *Hand1* allele allows only the expression of the *Hand1^PO4−^* allele and, remarkably, this improves the phenotype. *Prrx1-Cre;Hand1^fx/PO4−^* mice display a partially restored humerus and scapula in forelimbs (Fig. 6C) as well as femur, ilium, ischium and pubis within the hindlimbs (Fig. 6I) when compared with forelimbs (Fig. 6B) and hindlimbs (Fig. 6H) of *Prrx1-Cre;Hand1^+/PO4−^* mice. Hand2 is highly related to Hand1, can dimerize with it, regulates expression of *Shh* and occupies upstream regulatory sequences within proximal-anterior genes, repressing their expression (Galli et al., 2010; Firulli et al., 2000; Osterwalder et al., 2014). Intercross of *Hand2^neo/+^* onto the *Prrx1-Cre;Hand1^+/PO4−^* background also improves the development of affected limb structures (Fig. 6D,J). Reduction of *Hand2* dosage on the *Prrx1-Cre;Hand1^fx/PO4−^* background does not further improve the phenotype (Fig. 6E,K). *Prrx1-Cre;Hand2^neo/+^* mice, as expected, show no observable phenotype (Fig. 6F,L).

**Fig. 7.**
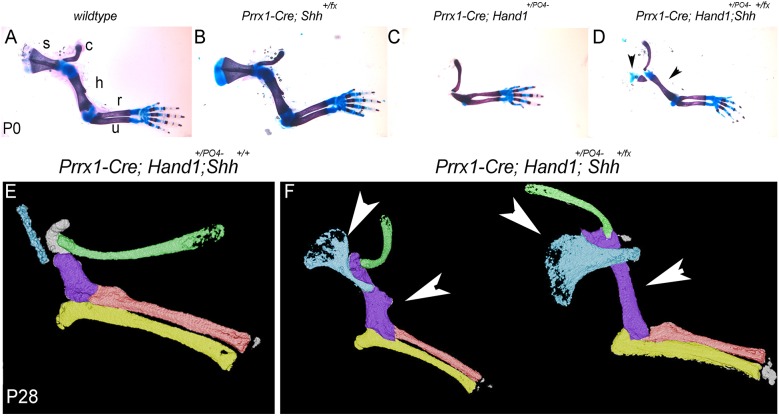
***Prrx1-Cre;Hand1^+/PO4−^* mutant limb structure is partially restored by lowering *Shh* gene dosage.** (A-D) P0 right forelimb skeletal preparations from (A) wild-type, (B) *Prrx1-Cre;Shh^fx/+^*, (C) *Prrx1-Cre;Hand1^+/PO4−^* and (D) *Prrx1-Cre;Hand1^+/PO4−^;Shh^fx/+^* mice. *Shh* heterozygosity shows no obvious phenotype. *Prrx1-Cre;Hand1^+/PO4−^* mice display a loss of scapula (s), reduced humerus (h) and smaller radius (r) and ulna (u). Reduction of Shh gene dosage partially improves humerus and scapula development (arrowheads). (E,F) Micro-CT analysis of P28 *Prrx1-Cre;Hand1^+/PO4−^* mutant (E) and two *Prrx1-Cre;Hand1^+/PO4−^;Shh^fx/+^* (F) mice. These older rescued animals show improvement to scapula (turquoise) and humerus (purple) development (arrowheads). *n*=5.

Given the reduced proximal-anterior marker expression (Fig. 5) combined with the expansion of *Shh* and *Gli1* and reduction of the transcriptional repressor *Gli3*, we reasoned that the *Prrx1-Cre;Hand1^+/PO4−^* mutant limb phenotype was also the result of expanded Shh-driven posterior gene regulatory networks, probably through augmenting the repressive functions of Hand2 (via dimerization) on proximal-anterior gene expression (Galli et al., 2010; Firulli et al., 2000; Osterwalder et al., 2014). Therefore, we intercrossed the *Shh* conditional allele onto *Prrx1-Cre;Hand1^+/PO4−^* mutants to determine if lowering the gene dosage of *Shh* would also restore anterior limb structures (Fig. 7). Results show that, when compared with wild type, *Prrx1-Cre;Shh^+/fx^* neonates exhibit no noticeable limb defects (Fig. 7A,B). As expected, *Prrx1-Cre;Hand1^+/PO4−^* neonatal limbs display loss of scapula and humerus, maintain the clavicle, with reduced size of radius and ulna (Fig. 7C). P0 *Prrx1-Cre;Hand1^+/PO4−^;Shh^+/fx^* mutants show a partial restoration in humerus and scapula size (Fig. 7D). A limited number of *Prrx1-Cre;Hand1^+/PO4−^* and *Prrx1-Cre;Hand1^+/PO4−^;Shh^+/fx^* mutants survived until P28. Micro-CT scans showed that some additional development occurs in the P28 *Prrx1-Cre;Hand1^+/PO4−^* mice, with some scapula and humerus development (Fig. 7E), while the *Prrx1-Cre;Hand1^+/PO4−^;Shh^+/fx^* mutants displayed a marked improvement of these structures (Fig. 7F). This supports the idea that mutant phenotypes result from the disruption of anterior gene regulatory networks and the expansion of posterior gene expression, at least in part through enhanced Hand2 transcriptional repression.

## DISCUSSION

Limb morphogenesis is a complex process that allows for significant variation in structure leading to diversity within a population that is tested by natural selection. Indeed, the high incidence of limb congenital abnormalities supports this idea. It is now established that both anterior and posterior signaling networks, which integrate patterning information, guide the forming limb (Galli et al., 2010; Osterwalder et al., 2014). The interlinking proximal-anterior gene regulatory networks within the forming limb are initiated in part by expression of Hand2, which activates Shh signaling in the posterior domains (Galli et al., 2010; Osterwalder et al., 2014). The anterior gene regulatory program is maintained by expression of the repressor Gli3 in conjunction with Irx3/5 (Li et al., 2014) and Sall4 [within hindlimbs (Akiyama et al., 2015)]. This study shows that regulation of the bHLH dimer pool within the forming limbs influences the proximal-anterior gene regulatory network in both fore- and hindlimbs. Although Hand1 is not required for normal limb morphogenesis as determined by conditional deletion of the wild-type *Hand1* alleles using *Prrx1-Cre*, when Hand1 is transgenically expressed (Fig. S1) or when Hand1 dimer control via phosphoregulation of key threonine and serine residues present in all Twist family bHLH factors is dysregulated, gain-of-function phenotypes are encountered (Firulli et al., 2005, 2007, 2014). Most evident in the observed phenotypes is widespread cell-autonomous and non-cell-autonomous death in *Prrx1-Cre;Hand1^+/PO4−^* mutant limbs (Fig. 3). The robust cell dropout observed is consistent with the reduction in size observed in proximal limb structure. Although we observe specific changes in gene expression, the caveat of widespread cell death must be considered when interpreting the gene expression data. Interestingly, the related bHLH factor Hand2 limb loss-of-function mutants also exhibit significant cell death within the developing limb (Galli et al., 2010), underscoring the importance of maintaining balance within these gene networks.

Hand2 directly regulates limb *Shh* expression and directly inhibits expression of the proximal-anterior genes *Irx3*, *Irx5*, *Gli3* and *Alx4* (Galli et al., 2010; Osterwalder et al., 2014). Given that Hand1 phospho-mutants exhibit an increase in the Shh responder *Gli1*, a decrease in the Shh pathway inhibitor *Gli3*, and downregulation of *Irx3*, *Irx5* and *Alx4* (Fig. 5), we conclude that Hand1 dimer mutants enhance the established inhibitory functions of Hand2 on these proximal-anterior genes. Indeed, the observed Hand1 mutant phenotypes are partially rescued by lowering either *Hand2* or *Shh* gene dosage (Figs 6 and 7). It is likely that Hand1 dimer changes increase the efficacy by which Hand2 represses this gene network. Hand2 dimer regulation is also influenced by Twist1 interactions during limb morphogenesis, resulting in SCS when dysregulated (Firulli et al., 2005, 2007). This study adds the influence of *Hand1* gene expression to the more proximal limb structures, not via direct loss-of-function effects but by augmenting the bHLH dimer pool within the limb mesenchyme affecting Hand2 function. Thus, it is not only the level of transcription and the spatial domain of expression that are critical for normal development, but bHLH phosphoregulation is also crucial and, in the case of Hand1, more so than its complete loss-of-function.

Interesting and confusing is the observation that removing the wild-type *Hand1* allele (*Prrx1-Cre;Hand1^fx/PO4−^*) also improves the phenotype. We observed the same phenomena when *Hand1^fx/PO4−^* and *Hand1^fx/PO4+^* mutants are expressed within neural crest cells (Firulli et al., 2014). These observations suggest that by expressing both the mutant Hand1 and wild-type Hand1 protein within the same cell, the wild-type Hand1 is contributing to the observed deleterious functions. Dimerization of the mutant Hand1 could sequester or free up a key bHLH partner that dimerizes inappropriately with wild-type Hand1 or allow for increased Hand2 activity. It is also possible that Hand1-Hand2 dimers themselves repress the proximal-anterior genes more efficaciously. Indeed, the interdependence of Twist family bHLH factors is well established. Twist1 and Hand2, for example, have an antagonistic relationship during limb development via a dimer choice-driven mechanism that underlies the cause of SCS, a phenotype that in mice can be rescued by rebalancing *Hand2* and *Twist1* gene dosage (Firulli et al., 2005). Moreover, transgenic expression of tethered Twist1 and Hand2 dimers results in distinct limb phenotypes (Firulli et al., 2007). Our observations in this study are consistent with this dimer choice model.

*Shh* and *Hand2* expression in Hand1 phospho-mutants is not significantly altered (Fig. 4, Fig. S4). These results suggest that Hand1 dimer mutants are not influencing posterior limb gene expression directly. The anterior genes *Alx4*, *Irx3*, *Irx5* and *Gli3* are all Hand2 targets and are markedly downregulated in *Prrx1-Cre;Hand1^fx/PO4−^* mutants (Fig. 5M). *Alx4* mutants display polydactyly and increased ectopic *Shh* expression, which reflects a posteriorization of the limb (Kuijper et al., 2005). Gli3 is post-translationally processed into a transcriptional repressor that is inhibited by Shh signaling (Ahn and Joyner, 2004). Low Gli3 allows for Gli1 expression and the implementation of Shh target gene expression. Indeed, loss of *Gli3* results in polydactyly via the expanded reach of Shh signaling (Akiyama et al., 2015; Lopez-Rios et al., 2012; te Welscher et al., 2002). Genetically, *Gli3* antagonizes *Hand2* (te Welscher et al., 2002) and the reduction of *Gli3* observed in the *Prrx1-Cre;Hand1^+/PO4−^* mutants further supports expanded proximal gene regulation within the developing limb linked to Hand2. *Irx3* and *Irx5* are also negatively regulated by the Shh/Hand2 pathway. Early expression of these factors is required to specify limb progenitors that give rise to the humerus/femur, radius/tibia and digit 1 (Li et al., 2014). Comparison of *Prrx1-Cre;Hand1^+/PO4−^* mutants with *Irx3;Irx5* mutants reveals a similarity in phenotypes; however, there are notable differences, including the presence of a largely normal tibia, an occasionally absent fibula, and patent digit 1 (Fig. 1) (Li et al., 2014). Whereas Li et al. (2014) conditionally knocked out both genes, in our study we only observe 40% reduction in *Irx3/5* expression (Fig. 5M). It is likely that the variations observed between the two models are influenced by this difference. Interestingly, *Prrx1-Cre;Hand1^+/PO4−^* mutants also show similar phenotypes to both femoral focal deficiency (D'Ambrosio et al., 2016) and phocomelia (Vargesson et al., 2015), which similarly show both pelvis and femur development variations.

Although it is well established that Twist family bHLH proteins exhibit regulation of function via dimer choice (Firulli et al., 2000, 2003, 2005, 2007, 2014; Martindill et al., 2007), understanding the exact nature of the specific dimer complexes that drive the temporal-spatial gene regulatory networks within the forming limbs remains elusive. The difficulty lies in the ability to utilize techniques such as fluorescence resonance energy transfer (FRET) *in vivo* under conditions in which genes are expressed at endogenous levels. To accomplish this would require a series of Hand1 and Hand1 dimer mutant YFP fusion proteins knocked into the endogenous locus complemented by a similar series of Hand1 dimer partner CFP fusion proteins knocked into their respective loci (*Twist1*, *Hand2*, *Tcf3*, and so on). Assuming that these dimer-reporter mice would be viable as homozygotes, one could assay endogenous changes in dimerization of two specific partners and these data could be cross-referenced to DNA occupancy data to complete a picture of specific Twist family dimer complexes driving gene regulatory networks.

## MATERIALS AND METHODS

### Mouse strains and genotyping

*Hand1^stopfloxHand1T107;S109A^* (*Hand1^PO4^*^−^) and *Hand1^stopfloxHand1T107;S109D^* (*Hand1^PO4^*^+^) mice were generated and genotyped as described (Firulli et al., 2014). B6.129S4-*Gt(ROSA)26Sortm1Sor*/J (*R26R^lacZ^*) mice were genotyped using a probe located 5′ of the Stop-Flox (and provided by Dr Phillippe Soriano, Mount Sinai Hospital, NY, USA). Both *Hand1^+/PO4^*^−^ and *Hand1^+/PO4^*^+^ alleles were bred onto a *R26R^lacZ^* homozygous background and females of this genotype were crossed to *Prrx1-Cre* or *Prrx1-Cre;Hand1^fx/fx^* males to generate either *Hand1^+/P^^O4^*^−^ and *Hand1^+/PO4+^* or *Hand1^fx/PO4^*^−^ and *Hand1^fx/PO4+^* embryos. *Hand2^n^^eo/+^* were genotyped as described (Srivastava et al., 1995). *Shh* conditional knockout mice (B6;129-*Shh^tm2Amc^*/J) were obtained from Jackson Labs and crossed with *Hand1^PO4^*^−^ mice. Both male and female mice/embryos are used in this study. Ages are indicated in figures. All animal experiments were performed in accordance with NIH guidelines (Guide for the Care and Use of Laboratory Animals) following the Indiana University Animal Care and Use Committee approved animal protocol 10809.

### *In situ* hybridization and qRT-PCR

Digoxygenin-labeled section and whole-mount *in situ* hybridizations were carried out as described (Firulli et al., 2010; Vincentz et al., 2008). qRT-PCR was performed on a QuantStudio 3 (Applied Biosystems) quantitative thermocycler using TaqMan primers (Life Technologies) recognizing the following transcripts: *Bmp10*, *Shh*, *Fgf8*, *Gli1*, *Pax9*, *Alx4*, *Irx3*, *Irx5* and *Gli3*. Forelimbs from viable embryos were isolated and stored in RNAlater (Invitrogen) for RNA isolation and genotyped from the yolk sac DNA. Total RNA was isolated using the High Pure RNA Tissue Kit (Roche) and cDNA was prepared using the High-Capacity cDNA Reverse Transcription Kit (Life Technologies) following the manufacturers' protocols. Error bars denote the maximum and minimum relative level of gene expression in the test samples calculated using the confidence level set in the QuantStudio 3 and 5 software analysis settings. Statistical significance was determined using Student's two-tailed *t*-test. *P*≤0.05 was regarded as significant. *n*≥5 was applied in all experiments to account for embryo viability and individual expression characteristics. Expression patterns were observed to be consistent within all viable replicates.

### Lysotracker cell death analysis

Lysotracker (Life Technologies) was incubated with embryos as per the manufacturer's instructions as described (Firulli et al., 2014). Embryos were imaged on a Zeiss Stemi SV 11 dissection microscope fitted with a fluorescent light source and filter cubes. Images were collected on the red channel and converted to grayscale in Adobe Photoshop.

### Micro-computed tomography (micro-CT), planar radiography and skeletal preparations

Limb morphology of P0 mice was assessed using the high-resolution desktop X-ray microtomography SkyScan 1172 imaging system (SkyScan, Kontich, Belgium) using methods similar to those described previously (Firulli et al., 2014). Mice were scanned with an isotropic voxel size of 8 µm, with an energy level of 50 kV, and an aluminum 0.5 mm filter. A lower energy source was used to capture regions of undermineralized bone. 2D cross-sectional grayscale slices from each limb (∼600-800 slices per limb) were reconstructed using NRecon reconstruction software (SkyScan). Reconstructed slices were saved as individual TIFF images and converted to a DICOM (Digital Imaging and Communications in Medicine) format. DICOM files were used to create 3D models using OsiriX version 5.6 imaging processing software for DICOM images (Medical Imaging Software, Los Angeles, CA, USA). All 3D images were created using identical grayscale thresholds, with scaling of each image conserved. Overlaying skeletal structures were removed using a bone removal tool to isolate fore- and hindlimb structures. *n*≥4 per genotype scanned and reconstructed. Measurements of bones were performed on all scanned limbs and significance (*P*≤0.05) determined by *t*-test. Whole-body planar anteroposterior X-rays were collected on a subset of preserved mouse carcasses as described previously (McAteer et al., 2010). Skeletal preparations were performed as described (Firulli et al., 2005, 2014).
